# Supplementary figures and images for: Methylation pattern of polymorphically imprinted nc886 is not conserved across mammalia
Source: PLoS One. 2022 Mar 16;17(3):e0261481. doi: 10.1371/journal.pone.0261481 (PMC8926257; doi:10.1371/journal.pone.0261481)

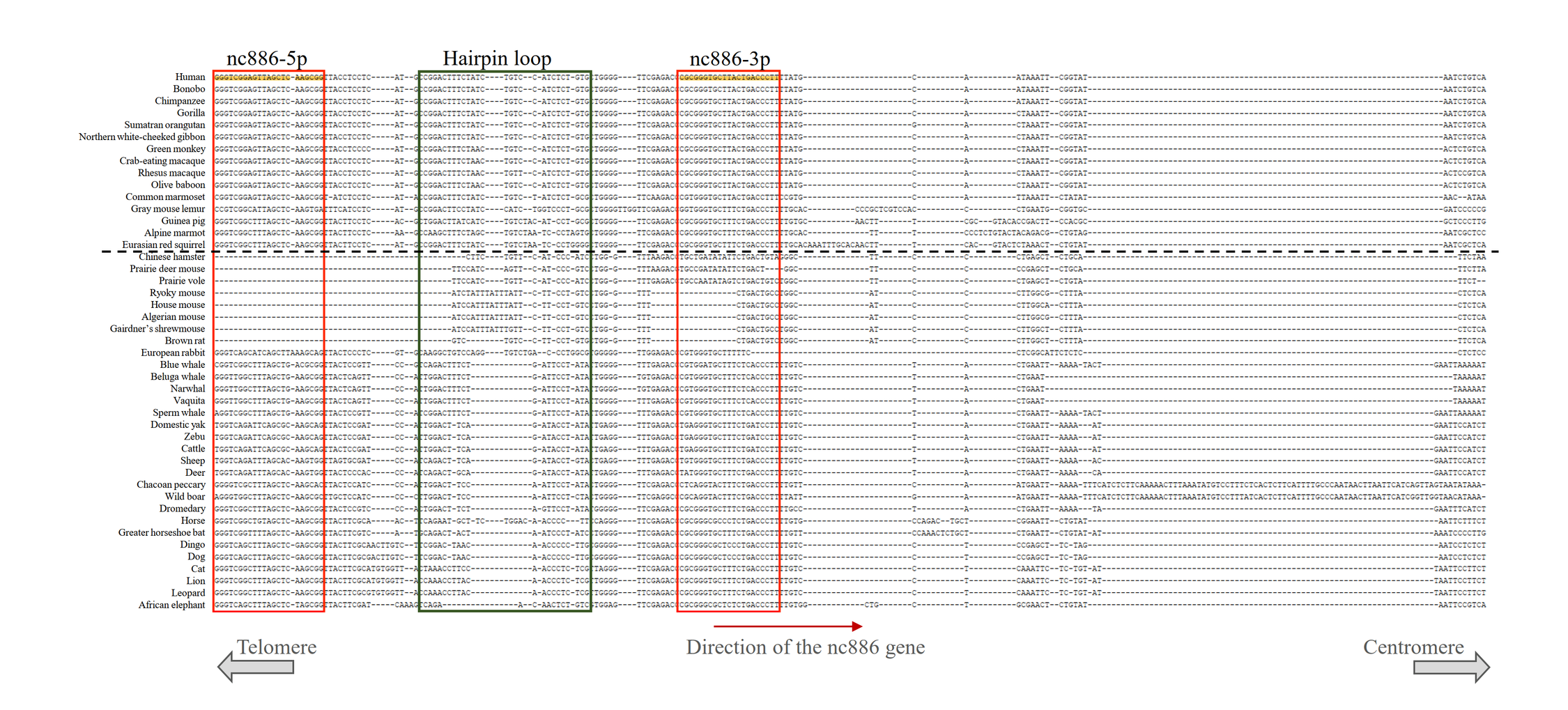

Supplement: S1 Fig — To state that a species presents the nc886 we required 80% sequence similarity with the human nc886 and presence of both nc886-5p and nc886-3p short RNAs and the hairpin loop present in the 102nt long nc886 RNA. Species above the dashed line were considered to harbour the full nc886 gene. Note: Sequence alignment figures are presented in the direction of the gene. Species with no identified alignment in this region were excluded from the figure (Sauropsids, opossum (Monodelphis domestica) and platypus (Ornithorhynchus anatinus)). (TIF) [file pone.0261481.s001.tif]

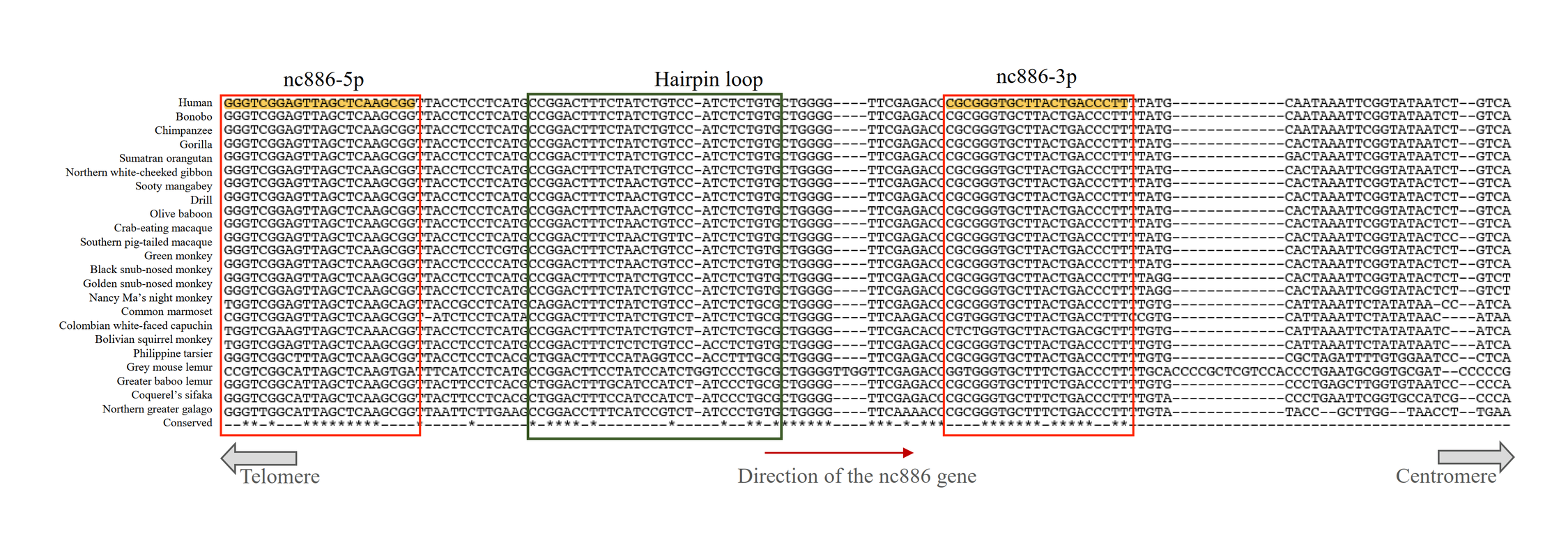

Supplement: S2 Fig — Sequence similarity to human nc886 gene decreases as evolutionary distance increases and greatest diverge is seen in the centromeric end of the gene. Note: Sequence alignment figures are presented in the direction of the gene. (TIF) [file pone.0261481.s002.tif]

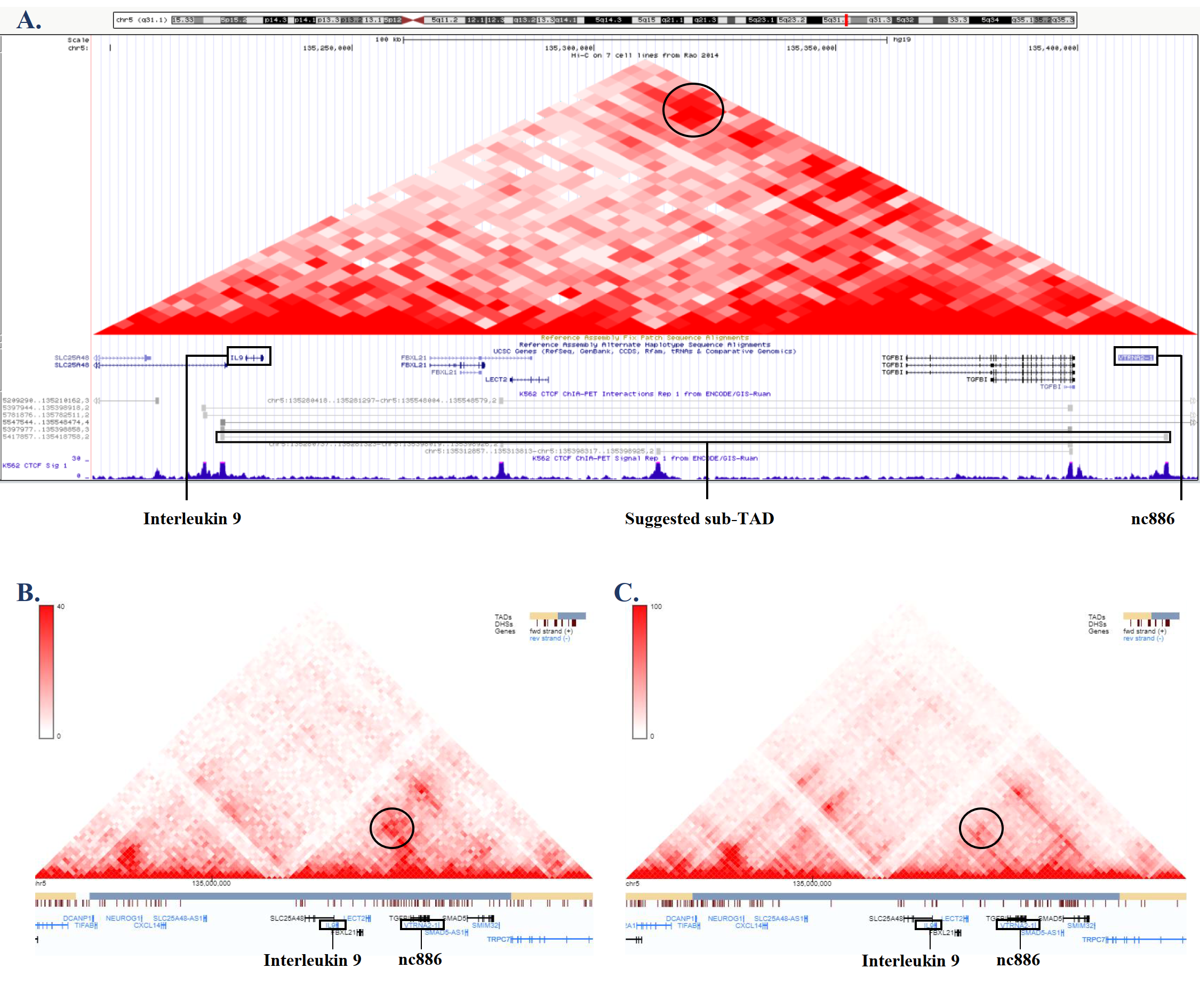

Supplement: S3 Fig — A) ChIA-PET data and in HI-C data in Genome browser cell lines and in 3D genome browser. B) HUVEC and C) K562 cell lines. The suggested sub-TAD has been indicated with a black square and interactions in HI-C data have been circumscribed. The telomeric CTCF binding site is located at chr5:135418124–135418523 and the CTCF binding site near IL9 at chr5:135223050–135223420. (TIF) [file pone.0261481.s003.tif]

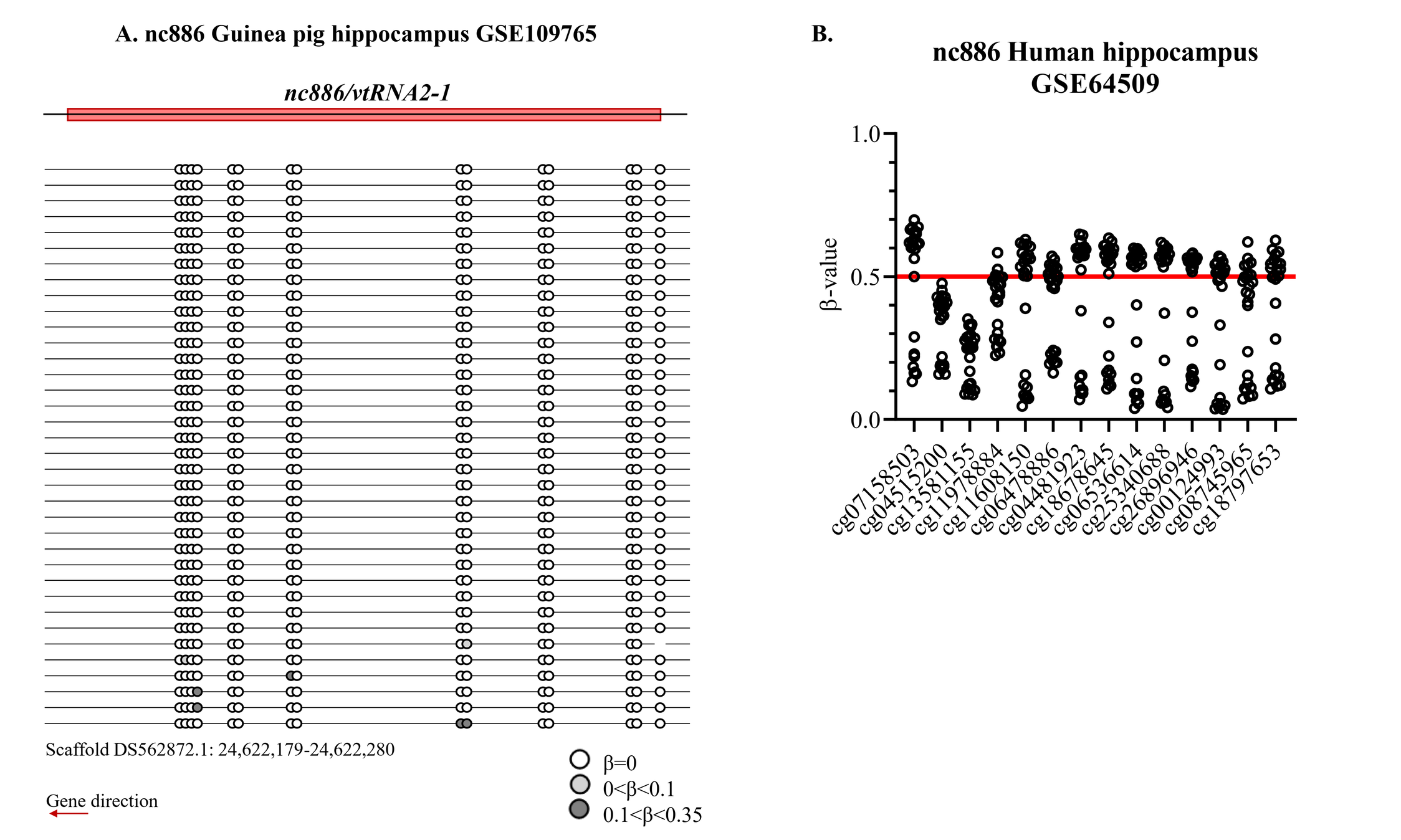

Supplement: S4 Fig — In (A) each circle presents a CpG site with measured methylation level and each line one individual guinea pig (n = 36). The number of reads per site is low (on average 7) but none of the samples provide data that would indicate anything but non-methylated DNA methylation status around the guinea pig nc886 gene. In human hippocampi (B) the methylation pattern of nc886 presents the expected binomial methylation pattern, which is in line with reported frequency of 25% non-methylated individuals and 75% individuals with monoallelic methylation in a population. (TIF) [file pone.0261481.s004.tif]

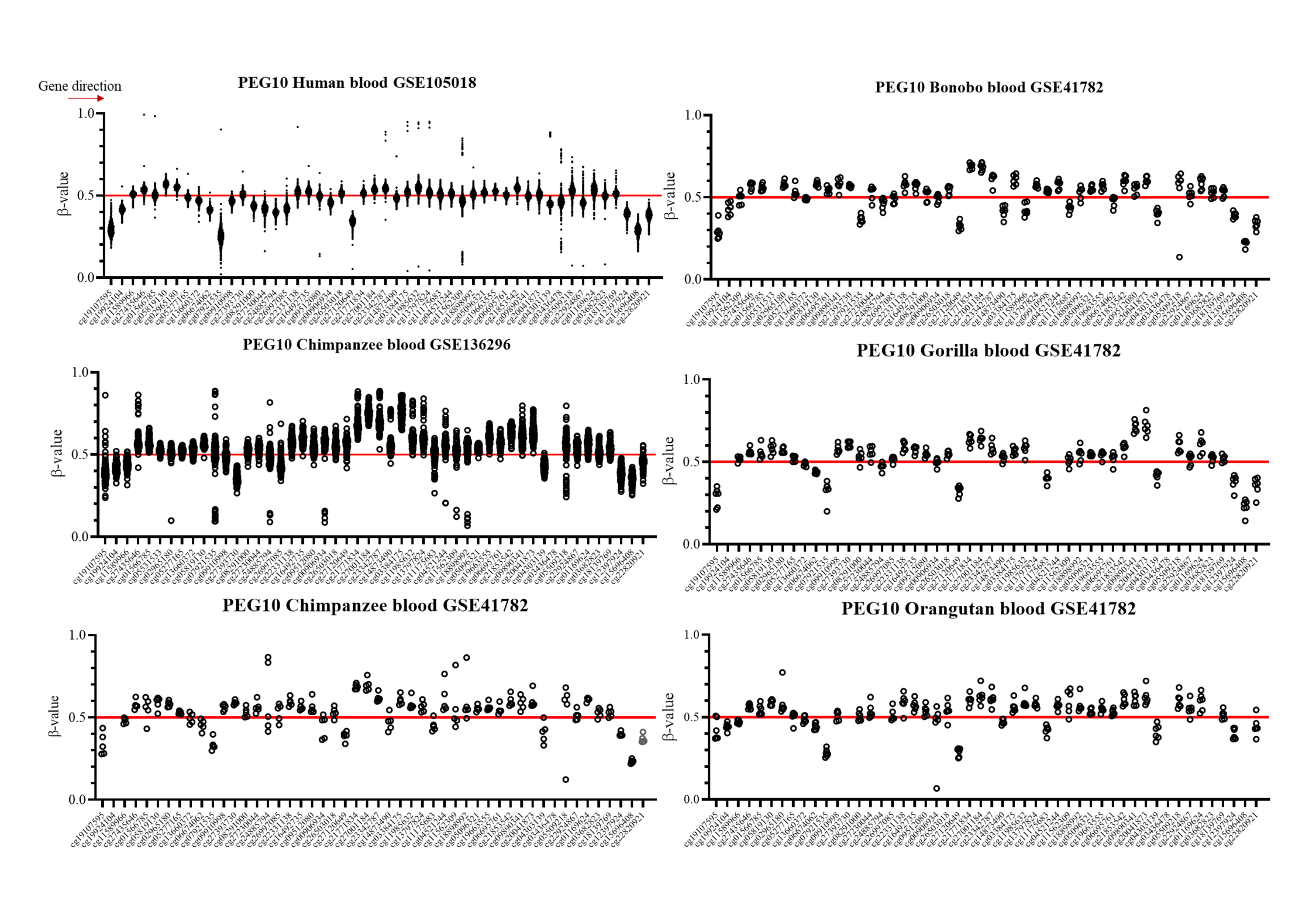

Supplement: S5 Fig — Only probes locating in sites with no clear sequence differences as compared to the human PEG10 sequence are shown. (TIF) [file pone.0261481.s005.tif]

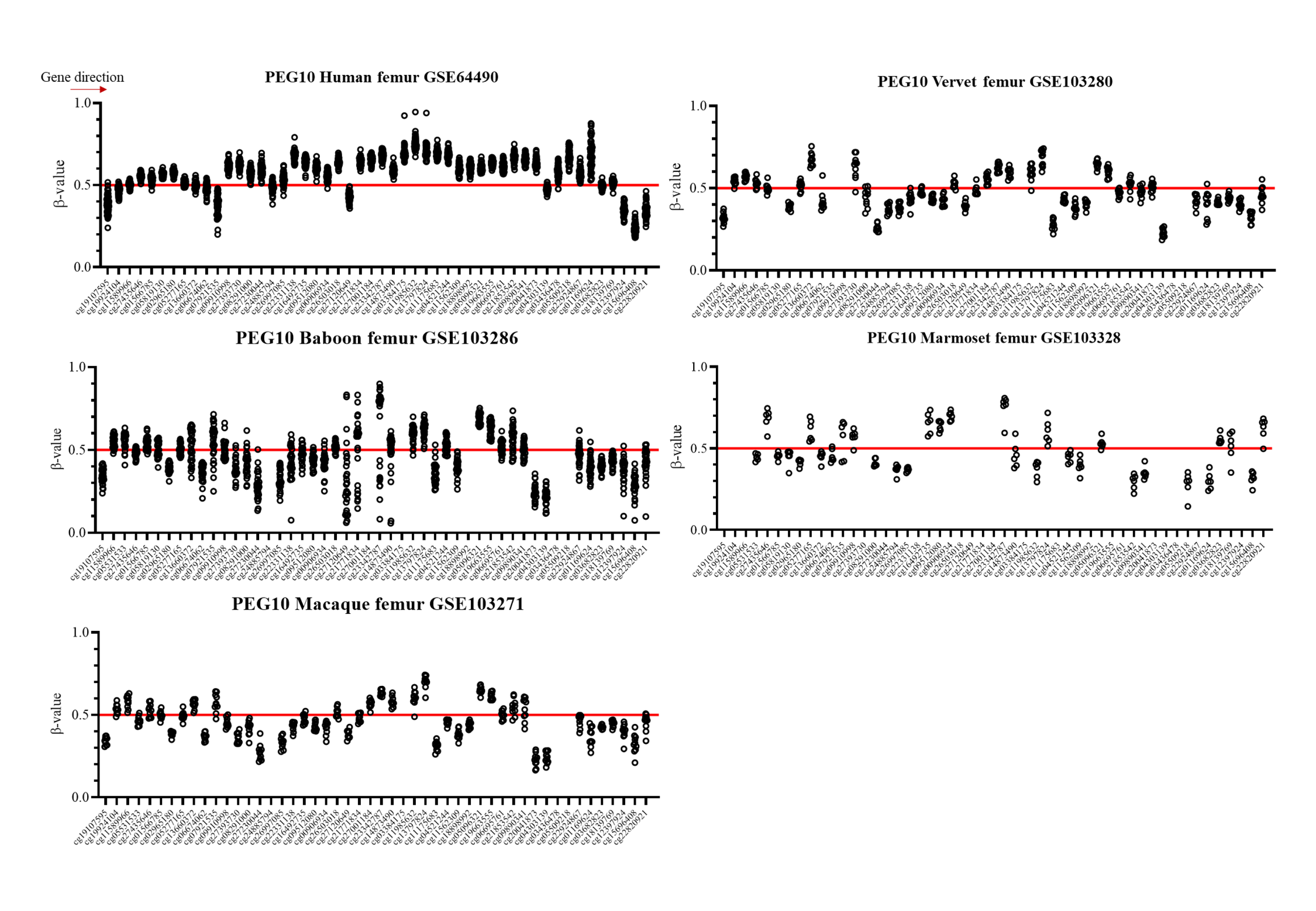

Supplement: S6 Fig — Only probes locating in sites with no clear sequence differences as compared to the human PEG10 sequence are shown. (TIF) [file pone.0261481.s006.tif]
